# The Electrical Review

**Published:** 1892-10

**Authors:** 


THE ELECTRICAL REVIEW has decided to add a department devoted to the application of electricity to medical treatment. The first instalment appeared September 10th, and will continue weekly. The price of *The Review* is $3.00 per year in advance. Sample copies sent on application. Address *The Electrical Review*, 13 Park Row, New York.

